# Rethinking causal assumptions about maternal BMI, gestational weight gain, and adverse pregnancy outcomes

**DOI:** 10.1186/s12916-024-03410-2

**Published:** 2024-05-15

**Authors:** Jodie M. Dodd, Jennie Louise, Andrea R. Deussen, Megan Mitchell, Lucilla Poston

**Affiliations:** 1 Department of Obstetrics and Gynaecology, The Robinson Research Institute, The University of Adelaide, Adelaide, South Australia Australia; 2 Department of Obstetrics and Gynaecology, Women’s and Babies Division, The Women’s and Children’s Hospital, 72 King William Road, North Adelaide, South Australia 5006 Australia; 3 SAHMRI Women and Kids, South Australian Health and Medical Research Institute, Adelaide, South Australia Australia; 4 Women’s and Children’s Research Centre, Women’s and Children’s Hospital Research Network, Adelaide, South Australia Australia; 5 Women and Children’s Health and School of Life Course Sciences, King’s College London, London, UK

**Keywords:** Gestational weight gain, Pregnancy, Dietary and lifestyle intervention, Obesity, Causal pathways, Body mass index

## Abstract

**Background:**

The aim of this study was to evaluate commonly assumed causal relationships between body mass index (BMI), gestational weight gain (GWG), and adverse pregnancy outcomes, which have formed the basis of guidelines and interventions aimed at limiting GWG in women with overweight or obesity. We explored relationships between maternal BMI, total GWG (as a continuous variable and as ‘excessive’ GWG), and pregnancy outcomes (including infant birthweight measures and caesarean birth).

**Methods:**

Analysis of individual participant data (IPD) from the i-WIP (International Weight Management in Pregnancy) Collaboration, from randomised trials of diet and/or physical activity interventions during pregnancy reporting GWG and maternal and neonatal outcomes.

Women randomised to the control arm of 20 eligible randomised trials (4370 of 8908 participants) from the i-WIP dataset of 36 randomised trials (total 12,240 women). The main research questions were to characterise the relationship between maternal BMI and (a) total GWG, (b) the risk of ‘excessive’ GWG (using the Institute of Medicine’s guidelines), and (c) adverse pregnancy outcomes as mediated via GWG versus other pathways to determine the extent to which the observed effect of maternal BMI on pregnancy outcomes is mediated via GWG. We utilised generalised linear models and regression-based mediation analyses within an IPD meta-analysis framework.

**Results:**

Mean GWG decreased linearly as maternal BMI increased; however, the risk of ‘excessive’ GWG increased markedly at BMI category thresholds (i.e. between the normal and overweight BMI category threshold and between the overweight and obese BMI category threshold). Increasing maternal BMI was associated with increased risk of all pregnancy outcomes assessed; however, there was no evidence that this effect was mediated via effects on GWG.

**Conclusions:**

There is evidence of a meaningful relationship between maternal BMI and GWG and between maternal BMI and adverse pregnancy outcomes. There is no evidence that the effect of maternal BMI on outcomes is via an effect on GWG. Our analyses also cast doubt on the existence of a relationship between ‘excessive’ GWG and adverse pregnancy outcomes. Our findings challenge the practice of actively managing GWG throughout pregnancy.

**Supplementary Information:**

The online version contains supplementary material available at 10.1186/s12916-024-03410-2.

## Background

Globally, rates of overweight and obesity continue to climb, affecting more than 1.46 billion adults [1] and 170 million children under 18 years [2]. There is increasing scientific data acknowledging that weight gain, overweight, and obesity have a highly complex aetiology, with multiple interacting (epi-)genetic and environmental causes fortified by neuroendocrine and immune responses [3]. However, the prevailing clinical narrative continues to attribute obesity and weight gain far more simplistically at an individual level, to eating more calories than are expended through physical activity, with a focus on individual responsibility to modify this imbalance [4].

The clinical narrative of pregnancy care similarly focuses on the simplistic energy in/energy out mismatch. Interventions have been designed to ensure optimal gestational weight gain (GWG), through dietary and physical activity modifications, with the intention of reducing adverse pregnancy outcomes. However, the aetiology of weight gain in pregnancy is more complex than simple energy imbalance. There exists significant interplay between physiological responses unique to pregnancy, all of which contribute to maternal fat deposition, plasma volume expansion, breast and uterine tissue hypertrophy, extracellular and amniotic fluid accumulation, and growth of placental and foetal tissue [5].

The Institute of Medicine (IOM) first released guidelines concerning appropriate GWG in 1990 [6], with a subsequent update in 2009 [7]. These recommendations advocate optimal GWG ranges, according to a woman’s body mass index (BMI) category, to reduce the risk of adverse pregnancy outcomes. Outcomes such as birth of an infant small (SGA) or large (LGA) for gestational age, caesarean birth, preterm birth, and postpartum weight retention have been documented to occur more frequently in women with GWG outside the recommended ranges (‘excessive’ or ‘inadequate’ GWG), and women in higher BMI categories are considered more likely to have ‘excessive’ GWG [7–9]. Systematic reviews of antenatal randomised trials (RCT) have therefore focused on dietary and activity modification to limit GWG in women with higher BMI; these have identified statistically significant but extremely modest changes in weight gain, ranging from 0.7 kg [10], 1.02 kg [11], up to 1.15 kg [12]. While some reviews have identified similarly modest reductions in pregnancy outcomes such as gestational diabetes [11, 12] and caesarean birth [11], these findings are not universal [10]. It is tempting to assume that achieving ‘optimal’ GWG (specifically avoiding ‘excessive’ GWG) is possible through lifestyle changes and that this will reduce adverse pregnancy outcomes (similar to the rhetoric that all weight loss is beneficial for health and within an individual’s control). There is, however, little biological plausibility or indeed evidence of causality to explain how such modest changes in weight during pregnancy could potentially impact clinical outcomes.

This study therefore considered the evidence for a relationship between maternal pre-pregnancy BMI, total GWG (as a continuous variable and as ‘excessive’ GWG), and adverse pregnancy outcomes. We utilised individual participant data (IPD) from the i-WIP (International Weight Management in Pregnancy) Collaboration [10] to explore:

1. The nature of the relationship between maternal BMI and GWG (as continuous variables);
2. The relationship between maternal BMI and risk of ‘excessive’ GWG (according to IOM guidelines);
3. The relationship between ‘excessive’ GWG and pregnancy outcomes; and
4. The extent to which GWG could be considered to mediate the relationship between maternal BMI and adverse pregnancy outcomes (including infant birthweight, birthweight *z*-score, LGA infant, and caesarean birth), such that it would be an appropriate target for intervention.

## Methods

### Quality assessment and study inclusion

We accessed individual participant data from the i-WIP Collaboration repository which has synthesised evidence from RCTs conducted globally of interventions of diet and/or physical activity during pregnancy on GWG and composite maternal and neonatal outcomes [10]. The original i-WIP dataset contained data on 12,240 women from 36 studies [10].

Individual studies were reviewed and excluded if data did not permit analysis of all randomised participants (i.e. missing data due to post-randomisation exclusions) to minimise potential for bias. Studies were also excluded if measures of relevant variables (BMI, GWG, mode of birth, gestational age at birth, infant birthweight) were either absent or inconsistent with measures defined in other studies. Cluster RCTs were eligible for inclusion, as were multi-arm trials. Data were analysed using Stata (v17) [13] and R (v4.0) [14]. Only data from the control groups were included, to avoid any intervention effects. Missing data for included studies were not imputed, as the IPD dataset did not contain auxiliary variables which were available and consistently defined in all studies. Our manuscript conforms with the AGReMA reporting guidelines [15].

### Statistical analysis

1. Relationship between maternal BMI and GWG

Regression analyses were employed to characterise the relationship between BMI and total GWG (with both as continuous variables); the aim was to properly describe the functional form of the relationship rather than to assess causality. Fractional polynomial modelling was used to assess nonlinearity, with the optimal model assessed using the deviance difference and by visual inspection of the resulting curves for biological plausibility. By these criteria, it was determined that a linear model was most appropriate. The initial models included interaction terms to allow for effect modification by parity (nulliparous vs multiparous) and maternal age; it was not possible to consider other potential confounders or effect modifiers as this resulted in the exclusion of too many studies missing the relevant variables. As the effect of maternal BMI on GWG was seen to vary by parity, the final model retained this interaction term. As the data were from multiple studies, and in order to ensure separation of between-study and within-study interaction effects, two-step IPD meta-analysis (IPDMA) methods were used, with sensitivity analysis performed using the one-step method [16] in order to obtain a pooled estimate of the mean change in total GWG corresponding to a change in maternal BMI. Between-study heterogeneity was assessed using the estimated ${\tau }^{2}$ as well as inspection of forest plots and Galbraith plots.

2) Risk of ‘excessive’ GWG and maternal BMI category

These analyses aimed to evaluate the impact of defining recommended GWG ranges by BMI category, given the observed continuous linear relationship in analysis 1. In particular, we hypothesised that the apparent increased risk of ‘excessive’ GWG for women with overweight or obesity might be explained partially or wholly by ‘step’ changes in ranges at BMI thresholds. Actual maternal BMI for each participant was rounded down to the nearest integer (e.g. 19.0 to 19.9 kg/m^2^ rounded to 19; 20.0 to 20.9 kg/m^2^ rounded to 20, etc.), to ensure the woman’s integer BMI value was within the correct BMI category and therefore the applicable GWG range as defined by the IOM recommendations. We therefore estimated the odds of ‘excessive’ GWG for maternal BMI categorised into 1 kg/m^2^ increments (i.e. 19.0–19.9; 20.0–20.9, etc.), by fitting logistic regression models with BMI considered a categorical variable. The odds of ‘excessive’ GWG were estimated separately for each BMI value and study-specific fixed intercepts were included to account for between-study heterogeneity.

3) Relationship between maternal BMI, ‘excessive’ GWG, and pregnancy outcomes

We next sought to examine the relationship between ‘excessive’ GWG and pregnancy outcomes at different values of maternal BMI. Specifically, we hypothesised that the use of BMI categories to define ‘excessive’ GWG would result in risks of GWG increasing at BMI thresholds, without a similar increase in the risk of adverse pregnancy outcomes, and that at the highest BMI values, the lower mean GWG would result in a lower risk of ‘excessive’ GWG while the risk of adverse outcomes was increased. In other words, the change in risk of ‘excess’ GWG would not covary with change in risk of adverse outcomes in a way which would be expected if ‘excessive’ GWG were on the causal pathway between maternal BMI and adverse outcomes. Rates of ‘excessive’ GWG were descriptively compared with pregnancy outcomes across maternal BMI (categorised into 1 kg/m^2^ increments as above), including mean infant birthweight, birthweight *z*-score, rates of caesarean birth, and rates of birth of an infant LGA.

4) Mediation analyses to determine the extent to which the effect of maternal BMI on pregnancy outcomes is mediated via GWG

Regression-based mediation analyses [17], with a logistic model for binary outcomes and linear regression for continuous outcomes, were undertaken to investigate the contribution of maternal BMI as a continuous variable to pregnancy outcomes and the extent to which the effect of maternal BMI was mediated via an effect on GWG as a continuous variable. For each outcome, the models allowed for exposure-mediator interaction (i.e. the mediating effect of GWG differed depending on maternal BMI and vice versa) [18]. The following estimates were then derived;

- BMI-GWG Interaction effect (exposure-mediator interaction);
- Natural direct effect: the effect on pregnancy outcomes that would result if we increased maternal BMI by 5 kg/m^2^ but held GWG constant;
- Natural indirect effect: the effect on pregnancy outcomes that would result if we held BMI constant but changed GWG to what it would have been with a 5 kg/m^2^ increase in BMI (roughly, the effect of BMI which is mediated via GWG); and
- Controlled direct effect at 6, 10, and 14 kg of GWG: the direct effect of BMI if we intervened such that all women had the relevant GWG. This effect will vary in the presence of exposure-mediator interaction.

**Fig. 1:**
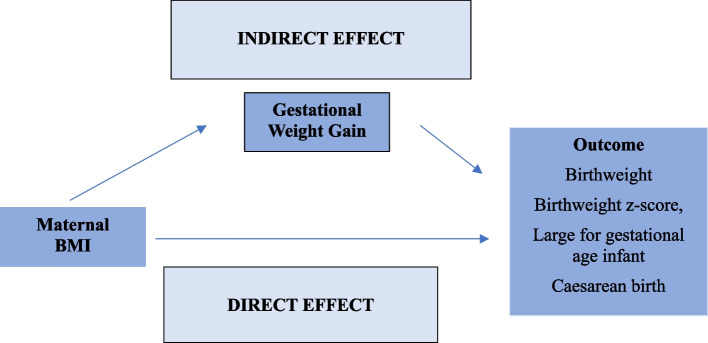
Schematic representation of mediation analysis indicating indirect and direct effects pathways for birthweight, birthweight z-score, large for gestational age infant, and caesarean birth

These relationships are schematically represented in Fig. 1. For a causal interpretation, the identification of these effects requires several assumptions, including no unmeasured confounding of the exposure-mediator (BMI-GWG) and exposure-outcome (BMI-outcome) relationships, and no confounding of the mediator-outcome (GWG-outcome) relationship that is affected by the exposure. As noted above, adjustment for potential confounders was limited by what was available in the dataset. However, specification of models for birthweight and caesarean birth took into account the fact that gestational age (GA) at birth was a confounder of the GWG-outcome relationship which could plausibly be affected by maternal BMI, by fitting a multiple-mediator model with GA as an additional mediator. For all outcomes, the mediation model was first fitted separately for each study, and standard errors were estimated using bootstrap resampling. Random effects meta-analysis was then used to combine the study-wise estimates. As with analysis 1, between-study heterogeneity was assessed using the estimated ${\tau }^{2}$ as well as forest and Galbraith plots.

We have complied with A Guideline for Reporting Mediation Analyses (AGReMA) and completed the AGReMA Checklist (Additional file 1: Table S1).

### Outcomes

Pregnancy outcomes examined in these analyses (birthweight, birthweight *z*-score, LGA and caesarean birth) were selected based on the outcomes of interest in the IOM recommendations and in subsequent observational and interventional studies targeting GWG as well as being the outcomes available (and consistently defined) in the i-WIP dataset. LGA infant (one of the components of the IOM composite adverse outcome) was defined as birthweight *z*-score > 90th percentile for GA. Birthweight *z*-score was defined using INTERGROWTH-21 standards [19] as implemented in the R package *growthstandards* [20]. Infant sex was not available for all observations, and where missing, *z*-scores were calculated by averaging male and female *z*-scores. The decision was made to examine birthweight and birthweight *z*-score in addition to LGA, as these are continuous measures and hence have greater statistical power than the dichotomised outcome; while birthweight *z*-score is preferable due to its being standardised to GA and sex, we examined birthweight as well due to the difficulties in ascertaining all information required to calculate *z*-scores. Caesarean birth was recorded for individual studies but was not differentiated by elective and emergency procedures.

### Patient involvement

Research participants contributed to the design of individual studies but were not involved in the development of this current research question.

## Results

### Study selection

**Fig. 2:**
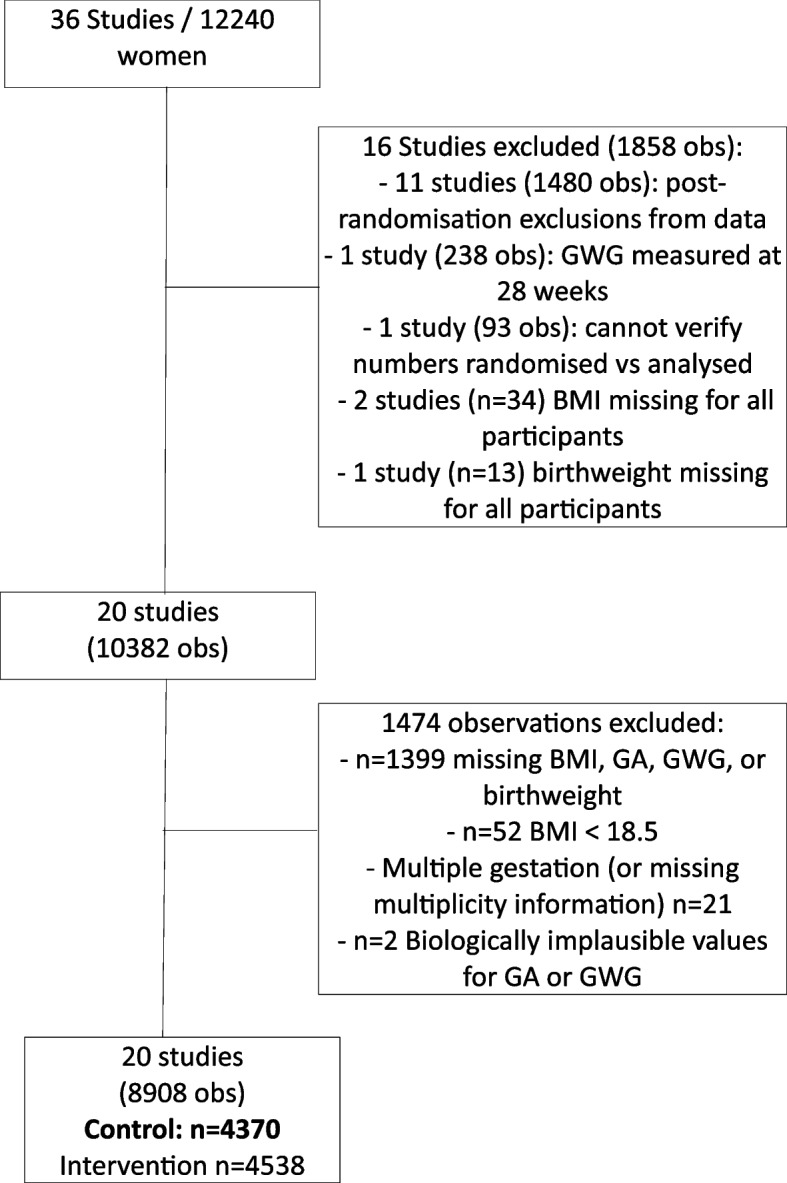
Flow of studies

The original i-WIP dataset contained data on 12,240 women from 36 studies [10]. Sixteen studies were excluded as outlined in Fig. 2 and Table 1. Two cluster RCTs [21, 22] and two multi-armed studies [23, 24] were included. In total, data from 20 RCTs, involving 8908 women, of whom 4370 were randomised to the control group were included (Table 2). The baseline characteristics of the included participants are presented in Table 3.

**Table 1:** Excluded studies

Study	Control	Intervention	Reason excluded
Barakat 2008 [25]	70	72	Post-randomisation exclusions from dataset (participants who discontinued intervention)
Barakat 2011 [26]	30	34	Post-randomisation exclusions from dataset (participants who discontinued intervention)
Barakat 2012 [27]	151	137	Post-randomisation exclusions from dataset (participants who discontinued intervention + others)
El Beltagy 2013 [28]	40	46	Abstract only (no article), cannot verify randomisation/numbers
Guelinckx 2010 [29]	53	55	Post-randomisation exclusions from dataset (‘dropouts’, participants developing GDM)
Harrison 2013 [30]	96	104	GWG was measured at 28 weeks
Hui 2011 [31]	88	102	Post-randomisation exclusions from dataset (participants who discontinued intervention)
Khaledan 2010 [32]	21	18	Post-randomisation exclusions from dataset (GDM)
Ong 2009 [33]	6	7	Birthweight missing for all participants
Oostdam 2012 [34]	39	41	Post-randomisation exclusions from dataset (participants who discontinued intervention or developed blood glucose > 6)
Perales 2015 [35]	73	90	Post-randomisation exclusions from dataset (participants who discontinued intervention + others)
Perales 2016 [36]	83	83	Post-randomisation exclusions from dataset (participants who discontinued intervention + others)
Prevedel 2003 [37]	19	22	Post-randomisation exclusions from dataset (participants who discontinued intervention + others)
Wolff 2008 [38]	30	26	Post-randomisation exclusions from dataset (‘dropouts’, participants developing GDM)
Yeo 2000 [39]	8	8	BMI missing for all participants
Yeo unpub [40]	12	6	BMI missing for all participants

**Table 2:** Included studies

Study	Control	Intervention
Althuizen 2013 [41]	99	94
Baciuk 2008 [42]	37	32
Bogaerts 2013 [23]	138	58
Dodd 2014 [43]	870	897
Haakstad 2011[44]	40	41
Jeffries 2009 [45]	110	122
Khoury 2005 [46]	102	94
Luoto 2011 [21]	165	215
Nascimento 2011 [47]	40	37
Petrella 2014 [48]	28	30
Phelan 2011 [49]	193	190
Poston 2015 [50]	619	576
Rauh 2013 [22]	77	150
Renault 2014 [24]	132	244
Ruiz 2013 [51]	457	470
Sagedal 2017 [52]	288	291
Stafne 2012 [53]	340	385
Vinter 2011 [54]	148	144
Vitolo 2011 [55]	152	149
Walsh 2012 [56]	335	319

**Table 3:** Baseline characteristics of included participants

Characteristic	Control	Intervention	Overall
Number of participants	4370	4538	8908
BMI (kg/m^2^): mean (SD)	29.28 (6.60)	29.12 (6.70)	29.20 (6.65)
BMI category: *N* (%)			
18.5–24.9	1360 (31.12)	1490 (32.83)	2850 (31.99)
25.0–29.9	1142 (26.13)	1158 (25.52)	2300 (25.82)
≥ 30.0	1868 (42.75)	1890 (41.65)	3758 (42.19)
Parity: *N* (%)			
0	2152 (49.24)	2292 (50.51)	4444 (49.89)
1 +	2213 (50.64)	2245 (49.47)	4458 (50.04)
Missing	5 (0.11)	1 (0.02)	6 (0.07)
Maternal age: mean (SD)	29.98 (5.19)	30.10 (5.08)	30.04 (5.13)
Smoking: *N* (%)			
Non-smoker	3339 (76.41)	3535 (77.90)	6874 (77.17)
Smoker	682 (15.61)	661 (14.57)	1343 (15.08)
Missing	349 (7.99)	342 (7.54)	691 (7.76)

### Findings

1. Relationship between maternal BMI and GWG

**Fig. 3:**
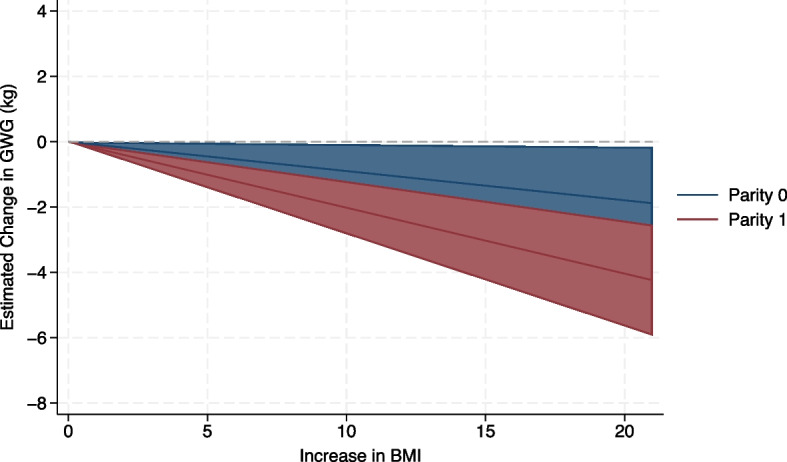
Estimated change in mean gestational weight gain with increasing maternal body mass index, for nulliparous and multiparous women. Estimated effect showing the difference in effect between nulliparous (parity 0; blue line) and multiparous (parity ≥ 1; red line) women. The deviation from the dotted line represents the extent to which gestational weight gain (GWG) decreases in multiparous women and nulliparous women as body mass index (BMI) increases

As noted above, the relationship between maternal BMI and GWG was approximately linear: specifically, GWG decreased on average with increasing maternal BMI (Fig. 3). The relationship was stronger in multiparous women (estimated mean difference (EMD) − 1.01 (95% confidence intervals (CI) − 1.41 to − 0.61), compared with nulliparous women (EMD − 0.45 (95% CI − 0.86 to − 0.04), *p* = 0.03, respectively), that is, for every 5 kg/m^2^ increase in maternal BMI, mean GWG was reduced by 1.01 kg (between 0.6 and 1.41 kg) for multiparous women and by 0.45 kg (between 0.04 and 0.86 kg) for nulliparous women (Fig. 3).

2) Risk of ‘excessive’ GWG and maternal BMI category

**Fig. 4:**
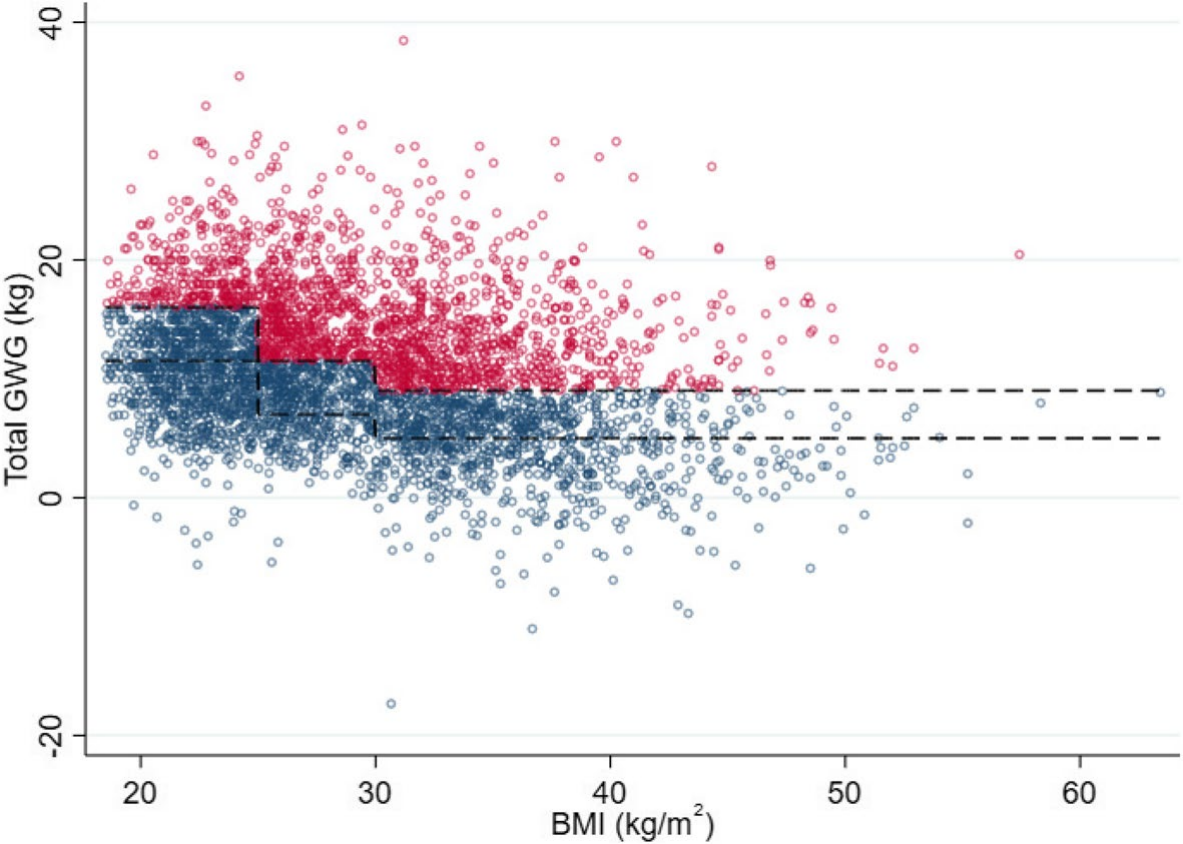
Graphical presentation of total GWG by maternal BMI with superimposed IOM recommended GWG ranges. The dashed lines indicate IOM recommended GWG ranges. ‘Excessive’ gestational weight gain (above IOM recommended range) is coloured red; ‘acceptable’ gestational weight gain (within IOM recommended range) is coloured blue

The estimated proportion of women with ‘excessive’ GWG did not increase linearly with maternal BMI. As shown in Fig. 4, there was a sharp increase in risk evident across the threshold between normal and overweight BMI categories and across the threshold between overweight and obese BMI categories, that is, the proportion of women with ‘excessive’ GWG increased from 20% (95% CI 15 to 25%) at BMI 24 kg/m^2^ to 54% (95% CI 49 to 59%) at BMI 25 kg/m^2^ and from 43% (95% CI 36 to 50%) at BMI 29 kg/m^2^ to 57% (95% CI 51 to 63%) at BMI 30 kg/m^2^. Furthermore, the risk of ‘excessive’ GWG within BMI categories tended to decrease with increased BMI.

3) Relationship between ‘excessive’ GWG and pregnancy outcomes

**Fig. 5:**
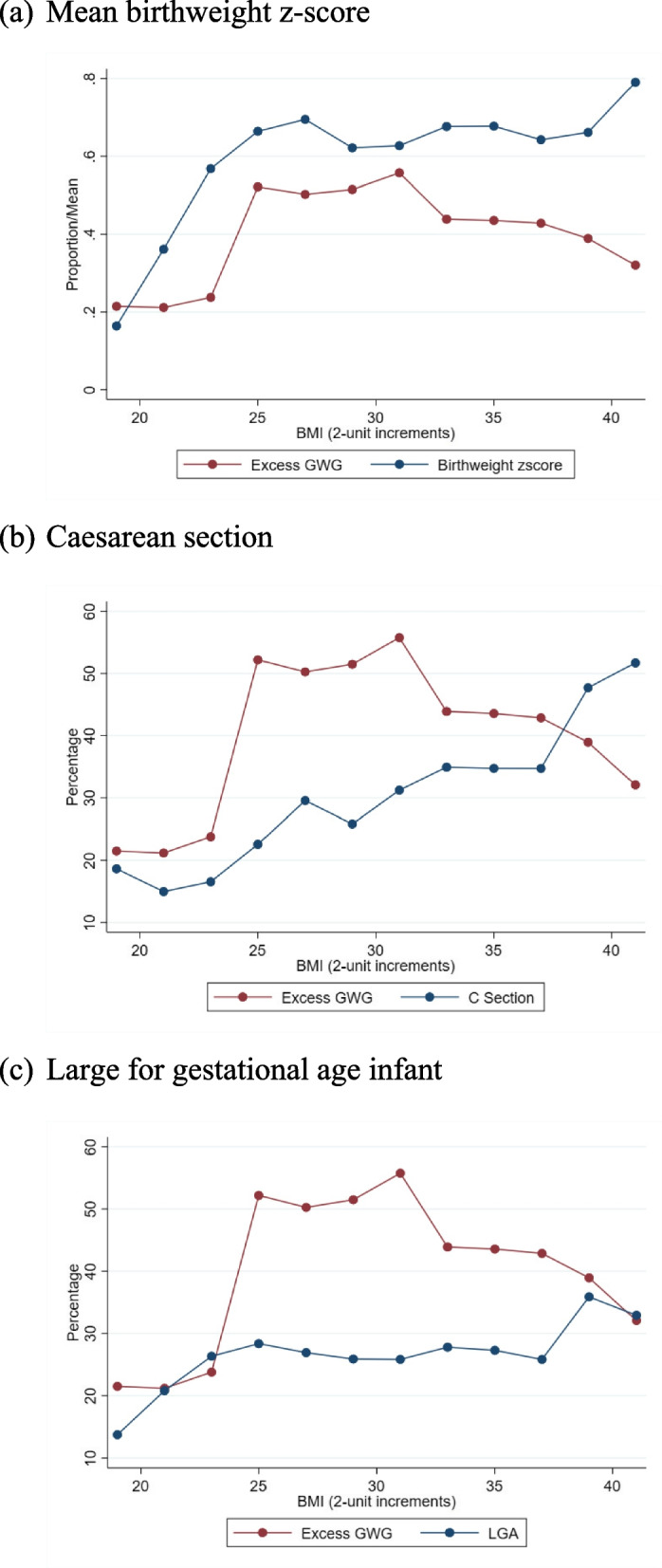
Rates of ‘excessive’ GWG for increases in maternal BMI against **a** mean birthweight *z*-score, **b** caesarean section, and **c** large for gestational age infant

Figure 5a–c shows the rate of ‘excessive’ GWG alongside pregnancy outcomes (mean birthweight/birthweight *z*-score, and rates of LGA/caesarean birth) by maternal BMI. The rates of adverse outcomes (or mean birthweight/birthweight z-score) did not covary with rates of ‘excessive’ GWG in a way which would be expected for a causal relationship. While adverse outcomes generally increased with higher maternal BMI, there were no sharp increases in risk at BMI category thresholds. Furthermore, at higher BMIs, rates of ‘excessive’ GWG decreased while the rates of adverse outcomes increased.

4) Mediation analyses to determine the extent to which the effect of BMI on pregnancy outcomes is mediated via GWG

Table 4 summarises the effect estimates from mediation analyses, quantifying the effect of BMI (via non-GWG pathways) on pregnancy outcomes (direct effect), and the extent to which this effect was mediated via GWG (indirect effect). Across all outcomes, there was evidence of a positive direct effect of maternal BMI (via pathways other than GWG). Specifically, a 5 kg/m^2^ increase in maternal BMI was associated with an average increase in birthweight of 65.6 g (95% CI 46.1 to 85.0 g), in birthweight z-score of 0.14 standard deviation (SD) (95% CI 0.10 to 0.18), an increased odds of a LGA infant (OR 1.38; 95% CI 1.25 to 1.52), and an increased odds of caesarean birth (OR 1.39; 95% CI 1.27 to 1.52).

**Table 4:** Effect estimates mediation analyses on pregnancy outcomes

	**Mean difference (95% CI)**		**Odds ratio (95% CI)**	
**Estimate**	*Infant birthweight*	*Infant birthweight z-score*	*LGA infant*	*Caesarean birth*
BMI-GWG interaction^a^	− 0.34 (− 0.88 to 0.19)	− 0.00 (− 0.00 to 0.00)	1.00 (0.99 to 1.00)	1.00 (1.00 to 1.00)
Natural direct effect^b^	65.56 (46.15 to 84.97)	0.14 (0.10 to 0.18)	1.38 (1.25 to 1.52)	1.39 (1.27 to 1.52)
Natural indirect effect^c^	− 14.21 (− 30.61 to 2.20)	− 0.03 (− 0.04 to − 0.01)	0.96 (0.93 to 0.99)	0.99 (0.97 to 1.01)
Controlled direct effect at GWG 6 kg^d^	57.54 (30.41 to 84.67)	0.13 (0.08 to 0.18)	1.45 (1.29 to 1.64)	1.41 (1.26 to 1.59)
Controlled direct effect at GWG 10 kg^d^	47.84 (13.84 to 81.84)	0.11 (0.04 to 0.18)	1.46 (1.22 to 1.75)	1.37 (1.17 to 1.61)
Controlled direct effect at GWG 14 kg^d^	40.64 (− 1.50 to 82.77)	0.09 (0.00 to 0.18)	1.47 (1.15 to 1.88)	1.34 (1.10 to 1.65)

Regression-based mediation analyses to investigate the contribution of maternal BMI to pregnancy outcomes. Model included exposure-mediator interaction (i.e. that the effect of GWG may differ depending on maternal BMI and vice versa) and the following estimates; ^a^BMI-GWG interaction effect. ^b^Natural direct effect, corresponding to a 5 kg/m^2^ increase in maternal BMI, assuming that there was no effect via GWG. ^c^Natural indirect effect, the effect of BMI as mediated via GWG. ^d^Controlled direct effect at 6, 10, or 14 kg of GWG, where the unmediated effect of BMI on the outcome was determined if GWG were set to 6 or 10 or 14 kg. *GWG*, gestational weight gain

Of note, however, the effect of maternal BMI via GWG (indirect effect) was to decrease mean birthweight (EMD 14.2 g; 95% CI 2.2 to 30.6 g) and birthweight *z*-score (0.03 SD; 95% CI 0.01 to 0.04) and to decrease the odds of a LGA infant (OR 0.96; 95% CI 0.93 to 0.99), with no evidence of an effect on caesarean birth (OR 0.99; 95% CI 0.97 to 1.01) (Table 4).

For all outcomes considered, there was little evidence of an exposure-mediator (BMI-GWG) interaction, and the controlled direct effects were therefore approximately the same for different fixed GWG values (at 6.0, 10.0, and 14.0 kg respectively) (Table 4).

Because of the inability to consider or adjust for potential confounders, the assumptions may not be met for identification of these estimates as causal effects. However, the observed estimates are sufficient to provide evidence that the relationship between maternal BMI and adverse pregnancy outcomes is mostly via pathways other than GWG.

## Discussion

### Principal findings

Our study explored the relationship between BMI and GWG, whether ‘optimal’ GWG ranges based on BMI categories adequately captured this relationship, and whether the relationship between BMI and adverse pregnancy outcomes was substantially mediated via GWG. Our findings demonstrate a linear relationship between maternal BMI and GWG, whereby GWG decreases with increasing BMI. We also demonstrated a relationship between BMI and adverse pregnancy outcomes, whereby the risk of adverse pregnancy outcomes increased with increasing maternal BMI.

There was, however, no evidence that maternal BMI increased the risk of adverse outcomes by increasing GWG. Moreover, ‘excessive’ GWG, defined in relation to BMI categories, did not appropriately capture the underlying relationship between BMI and GWG and did not covary with risks of adverse pregnancy outcomes in a way that would be expected for a causal relationship, that is, ‘excessive’ GWG, as currently defined, is not a good indicator of the risk of adverse pregnancy outcomes. By way of example, if we consider two women whose pre-pregnancy BMI differs by 0.2 kg/m^2^, both with a total GWG of 12 kg, their risks of adverse pregnancy outcomes are unlikely to differ substantially. However, if one woman’s BMI is 24.9 kg/m^2^ and the other’s is 25.1 kg/m^2^, the same GWG is at the lower end of ‘appropriate’ for the first woman but is considered ‘excessive’ for the second woman.

Our analyses have not evaluated the existence of a relationship between GWG (or ‘excessive’ GWG) and adverse pregnancy outcomes but rather have investigated the extent to which GWG mediates the effect of BMI. This is because three of our four outcomes are related to infant birthweight measures, caesarean birth is also partially associated with birthweight, and total GWG includes foetal weight as one of its components. This means that any observed relationship cannot be causal by definition, and estimates of the magnitude of the relationship have no clear interpretation. For the same reason, the indirect effects in our mediation analyses could not be given a causal interpretation even if all non-confounding conditions were met. They nevertheless provide evidence that GWG is not a mechanism by which increased BMI contributes to adverse pregnancy outcomes. Furthermore, this suggests that GWG is not an appropriate target to disrupt the link between maternal BMI and adverse pregnancy outcomes.

### Strengths and weaknesses

A strength of our study is the use of individual participant data, involving 20 randomised trials conducted globally, involving 4370 women and their infants. This sample included well powered, prospective studies conducted world-wide with pre-specified inclusion criteria and where pregnancy weight change was measured by clinical or research staff. It also guarded against bias by excluding studies where post-randomisation exclusions had occurred. While the sample size might be considered small compared with other studies [57, 58], it does provide adequate statistical power to investigate the effects of interest. Our statistical methodology adhered to a robust, pre-specified analysis plan, with pre-specified criteria for model selection where this was not stipulated a priori.

While it was only possible to consider pregnancy outcomes related to infant birthweight measures and caesarean birth, these outcomes are consistent with the composite outcome considered in the IOM recommendations and the primary focus of many studies investigating ‘adverse outcomes’ related to GWG. Furthermore, these outcomes were consistently available and uniformly defined in the dataset. A limitation includes missing data on infant sex, limiting our ability to define birthweight *z*-score and LGA. However, our findings of this current study confirm those of our previously reported analysis of BMI and GWG [59] in which infant sex was available.

There are many other outcomes of relevance (for example, but not limited to preterm birth, gestational hypertension, pre-eclampsia, gestational diabetes, maternal emotional well-being).

A more general limitation is that data were not consistently available for variables which might have been considered as confounders of the relationships examined in the study. For the same reason, we were unable to evaluate relationships between maternal BMI, GWG, and other outcomes such as hypertension, pre-eclampsia, and gestational diabetes. Within the i-WIP dataset, these outcomes have been variously defined, reflecting the lack of consensus that exists internationally, particularly around varied criteria used for the diagnosis of gestational diabetes [60]. However, it was not our intention to identify all outcomes which might be related to GWG but rather to demonstrate that a set of common assumptions regarding the existence of causal relationships between BMI, GWG, and the outcomes we chose should be questioned. Our findings are consistent with a previous report in a smaller dataset, where little evidence of a mediating relationship between BMI, GWG, and pregnancy outcomes was identified [59].

A large IPD meta-analysis of data from 25 cohort studies across Europe and North America has investigated the predictive value of maternal GWG ranges on a wide range of pregnancy outcomes [57]. In considering a composite outcome of one or more ‘adverse pregnancy outcomes’, GWG and their ranges had limited predictability in identifying women with either the composite outcome [57] or individual components, such as pre-eclampsia, caesarean birth, or preterm birth [57].

A subsequent report from members of the same group utilised 14 studies from the research collaborative to consider a wide range of clinical pregnancy and perinatal outcomes. A causal role was attributed to maternal BMI and many of the identified outcomes, with the researchers identifying the need for pre-conception interventions [58]. Findings from the current manuscript are consistent with the findings from this larger study which utilised different methodologies [58] in attributing a causal relationship between maternal BMI and pregnancy outcomes.

## Conclusions

The very limited impact of interventions during pregnancy on both GWG and pregnancy outcomes [10–12] may be substantially explained by our findings suggesting that GWG in general, and ‘excessive’ GWG in particular, are not related to pregnancy outcomes in an appropriate way. In particular, the effects of BMI (whether causal or not) appear to operate via pathways other than GWG, and ‘excessive’ GWG does not properly capture whether a woman’s GWG is truly excessive, in the sense of increasing risk of adverse outcomes. Moreover, to the extent that there is an association between increased GWG and adverse outcomes, this is, at least for birthweight-related outcomes, not causal in nature but rather due to the fact that foetal weight is a component of GWG. When taken together with a large body of evidence showing that the effect of interventions on GWG is modest at best, this suggests that while healthy eating and physical activity in pregnancy is ‘always a good idea’ [61], a continued and relentless search for the ‘right’ intervention targeting a non-modifiable outcome such as GWG is not only a waste of scarce healthcare resources but is setting women up to fail.

Our findings do identify evidence for a strong relationship between maternal BMI and pregnancy outcomes, highlighting the importance of robust research to further elucidate the causal mechanisms underpinning this relationship, thereby identifying more promising targets for intervention: for example, randomised trials encouraging women to optimise their health prior to conception [62–64].

Our findings are contrary to the assumptions underpinning the vast literature concerning GWG. In the interest of furthering scientific and clinical knowledge, we challenge other researchers in the field to similarly question the assumed causal mechanisms operating between maternal BMI, GWG, and pregnancy outcomes, as we have.

### Supplementary Information

**Additional file 1: Supplementary Table 1.** A Guideline to the Reporting of Mediation Analyses (AGReMA) Checklist.

## Data Availability

The full dataset or its subset and technical appendix are available from the data custodian (Birmingham University) at imsr@contacts.bham.ac.uk. Access to the dataset is regulated by terms and conditions available on request. The presented data are anonymised, and risk of identification of individual participants is low.

## Abbreviations

BMI: Body mass index
CI: Confidence intervals
EMD: Estimated mean difference
GWG: Gestational weight gain
IOM: Institute of Medicine
IPD: Individual participant data
IPDMA: Individual participant data meta-analysis
i-WIP: International Weight Management in Pregnancy Collaboration
LGA: Large for gestational age
RCT: Randomised controlled trial
SD: Standard deviation
SGA: Small for gestational age
