# Supplementary material for: Rethinking causal assumptions about maternal BMI, gestational weight gain, and adverse pregnancy outcomes
Source: BMC Med. 2024 May 15;22:197. doi: 10.1186/s12916-024-03410-2 (PMC11094971; doi:10.1186/s12916-024-03410-2)
Supplement: Supplementary file 1 — Additional file 1: Supplementary Table 1. A Guideline to the Reporting of Mediation Analyses (AGReMA) Checklist. [file 12916_2024_3410_MOESM1_ESM.docx]

**SUPPLEMENTARY FILES:**

Supplementary Table S1: AGReMA checklist

| **Section/Topic** | **Item #** | **Item description** | **Where addressed in manuscript** |
| --- | --- | --- | --- |
| Objectives^a^ | 4 | State the objectives of the study specific to the mechanisms of interest. The objectives should specify whether the study aims to test or estimate the mechanistic effects | Background: pg 6-7  Methods: pg 9-11 |
| Effects of interest^a^ | 9 | Specify the effects of interest | Background: pg 6  Methods: pg 11  Results- Findings: pg 13-15 |
| Causal assumptions^a^ | 11 | Specify assumptions about the causal model | Methods: pg 12  Results- Findings: pg 13-15  Discussion: pg 18 |
| Measurement^a^ | 12 | Clearly describe the interventions or exposures, mediators, outcomes, confounders, and moderators that were used in the analyses. Specify how and when they were measured, the measurement properties, and whether blinded assessment was used | Methods: pg 10-11 |
| Statistical methods^a^ | 14 | Describe the statistical methods used to estimate the causal relationships of interest. This description should specify analytical strategies used to reduce confounding, model building procedures, justification for the inclusion or exclusion of possible interaction terms, modelling assumptions, and methods used to handle missing data. Provide a reference to the statistical software and package used | Methods- Quality assessment and study inclusion: pg 8  Methods- Statistical analysis: pg 8-11 |
| Participants^a^ | 17 | Describe baseline characteristics of participants included in mediation analyses. Report the total sample size and number of participants lost during follow-up or with missing data | Table 3 |
| Outcomes and estimates^a^ | 18 | Report point estimates and uncertainty estimates for the exposure-mediator and mediator-outcome relationships. If inference concerning the causal relationship of interest is considered feasible given the causal assumptions, report the point estimate and uncertainty estimate | Methods-statistical analysis: pg 10-11 |
| Limitations^a^ | 20 | Discuss the limitations of the study including potential sources of bias | Discussion: pg 17-18 |
| Interpretation^a^ | 21 | Interpret the estimated effects considering the study's magnitude and uncertainty, plausibility of the causal assumptions, limitations, generalizability of the findings, and results from relevant studies | Discussion: pg 17-19 |
